# Education Research: Entrustment and Simulated Performance of Neurocritical Care Advanced Practice Providers

**DOI:** 10.1212/NE9.0000000000200284

**Published:** 2026-01-08

**Authors:** Daniel S. Harrison, Christa O.'Hana S. Nobleza, Matthew Bevers, Sahar F. Zafar, Rashid A. Ahmed, Ariel Nowicki, Elizabeth O.'B. Woods, Kelly Peronti, Erica Perets, Erika Sigman, Catherine S.W. Albin

**Affiliations:** 1 Department of Neurology, Boston University School of Medicine, Boston, MA;; 2 Department of Neurology, University of Tennessee Health Science Center, Memphis, TN;; 3 Department of Neurology, Baptist Memorial Hospital, Memphis, TN;; 4 Department of Neurology, Mass General Brigham, Boston, MA;; 5 Department of Neurology, Harvard Medical School, Boston, MA;; 6 Department of Neurology, Mass General Brigham, Boston, MA;; 7 Department of Neurology, Grady Memorial Hospital, Atlanta, GA;; 8 Departments of Neurology and Neurosurgery, Emory University School of Medicine, Atlanta, GA

## Abstract

**Background and Objectives:**

Entrustable professional activities (EPAs) have recently been defined for neurocritical care (NCC) advanced practice providers (APPs). There is no available assessment measure for APPs in neurology or NCC with supporting validity evidence. We aimed to assess the relationship between supervisor-assessed entrustment, performance in simulated medical and neurologic emergencies, and self-assessed entrustment among NCC APPs.

**Methods:**

This was a simulation quality improvement study. Participants were NCC APPs at 5 academic medical centers in the United States between April and November 2024. Participants completed 2 simulated scenarios designed to assess performance in the management of medical and neurologic emergencies. The primary outcome was the significance of the relationship between checklist-based performance in simulated scenarios and supervisor-assessed entrustment among NCC APPs. Secondary outcomes were the agreement between supervisor-assessed and self-assessed entrustment among NCC APPs.

**Results:**

There was a significant positive association between physician supervisor–assessed entrustment and performance on the critical action checklist (r_s_ = 0.57, *p* = 0.002). There was no significant correlation between performance in the simulated scenarios and APP supervisor–assessed entrustment (r_s_ = −0.02, *p* = 0.952). There was no difference between overall EPA physician supervisor assessment, APP supervisor assessment, and self-assessment (5-point entrustment-supervision scale median [interquartile range (IQR)], 4 [3–5] vs 4 [4–5] vs 4 [4–4], *p* = 0.598). There was substantial agreement in 490 discreet physician and self-assessed EPAs (κ_w_ = 0.62). Among 322 individual EPAs that were self-assessed and assessed by an APP supervisor, there was fair agreement (κ_w_ = 0.38). There was a significant positive association between physician supervisor–assessed entrustment and participant NCC experience (r_s_ = 0.85, *p* < 0.001).

**Discussion:**

Physician supervisor EPA-based assessment was positively correlated with NCC APP performance in 2 simulated neurologic and medical emergencies, providing validity evidence for the use of EPA-based assessments as a component of competency-based medical education. There was substantial agreement between physician supervisor and APP self-assessment of EPA-based entrustment, highlighting the potential to incorporate self-assessment in the planning of NCC APP onboarding and ongoing educational curricula.

## Introduction

Entrustable professional activities (EPAs) are units of professional practice that can be entrusted to an individual once they have attained sufficient, specific competence.^1^ These could include, for example, “Prioritize a differential diagnosis following a clinical encounter” or “Document a clinical encounter in the patient record.”^2^ They have been proposed as a useful concept in neurology education and were recently defined for neurocritical care (NCC) advanced practice providers (APPs), including physician assistants and nurse practitioners.^3,4^ EPAs were designed to guide curricular development and can be used for assessment.^1^ Many “entrustment-supervision scales” have been developed, each of which are supported by varying degrees of context-specific validity evidence.^5^ For example, level of entrustment has been shown to be strongly correlated with surgical performance in simulated scenarios.^6^ Outside the surgical setting, entrustment correlates with experience, although its relation to performance has not been assessed.^7^ While EPA-based assessment is in place for medical students and milestone-based assessment is commonly used for neurology residents and fellows, there is no commonly available assessment measure for APPs in neurology or NCC. As such, our primary aim was to assess the relationship between checklist-based performance in simulated medical and neurologic emergencies and supervisor-assessed entrustment among NCC APPs.

While supervisors are generally responsible for making decisions about the level of supervision a clinician needs to perform an activity, there are advantages to EPA self-assessment. Self-assessment promotes continuous learning and may increase one's engagement or investment in their training.^8^ It also could partially alleviate the burden of assessment on supervising clinicians.^9^ However, these putative benefits critically depend on accurate EPA self-assessment. Surgical resident EPA self-assessments have been shown to moderately agree with faculty assessments.^10^ Whether APPs or other neurology or NCC clinicians can provide accurate EPA self-assessments is unclear. As such, we further aimed to assess the agreement between supervisor-assessed and self-assessed entrustment among NCC APPs.

## Methods

This was a multicenter, quality improvement simulation study. Participants were NCC APPs at Brigham and Women's Hospital, Massachusetts General Hospital, Emory University Hospital, Grady Memorial Hospital, and Boston Medical Center between April and November 2024.

### Conceptual Framework

The study was based on the EPA framework. EPAs are units of professional practice that can be entrusted to an individual once they have attained sufficient, specific competence.^1^ They were originally developed as a framework for curricular development and can be used as an assessment tool. Ultimately, entrustment is binary (either an assessor trusts a learner to perform an activity or does not). However, when used as a tool for feedback or assessment, entrustment may be broken down into more nuanced levels of supervisory need with qualifiers.^3^ For example, a learner may not be entrusted to perform an activity at all (i.e., shadowing); be entrusted to perform an activity with direct, proactive supervision; be entrusted to perform an activity with indirect supervision immediately or remotely available; or be entrusted to supervise others performing the activity. Messick's unified validity framework was used in the collection of validity evidence for EPA-based assessments, simulation scenarios, and behavior checklists in this context.^11-13^ Methods used to demonstrate content validity, response process validity, internal structure, and relationship to other variables are summarized in Table 1.

**Table 1:** Validity Evidence for Entrustable Professional Activity–Based Assessments, Simulated Scenarios, and Behavior Checklists

Category	Evidence for validity in this context
Content	EPA-based assessment: survey items derived from expert consensus entrustable professional activities Simulation scenarios: iteratively developed by content experts, reviewed by outside subject matter expert, pilot tested, and revised Behavior checklist: iteratively developed by content experts based on peer-reviewed consensus guidelines, reviewed by outside subject matter experts, pilot tested, and revised
Response process	EPA-based assessment: survey questions followed best practices for entrustment-supervision scale item formulation Simulation scenarios: video capture for quality control, participant confidentiality requested for assessment security/prevention of cheating Behavior checklist: raters trained and calibrated before scoring participant videos
Internal structure	EPA-based assessment: Cronbach α Behavior checklist: interrater reliability in pilot test scoring
Relationship to other variables	Associations between entrustment, checklist-based simulated performance, and clinician experience

Abbreviation: EPA = entrustable professional activity.

### Study Protocol

**Figure 1:**
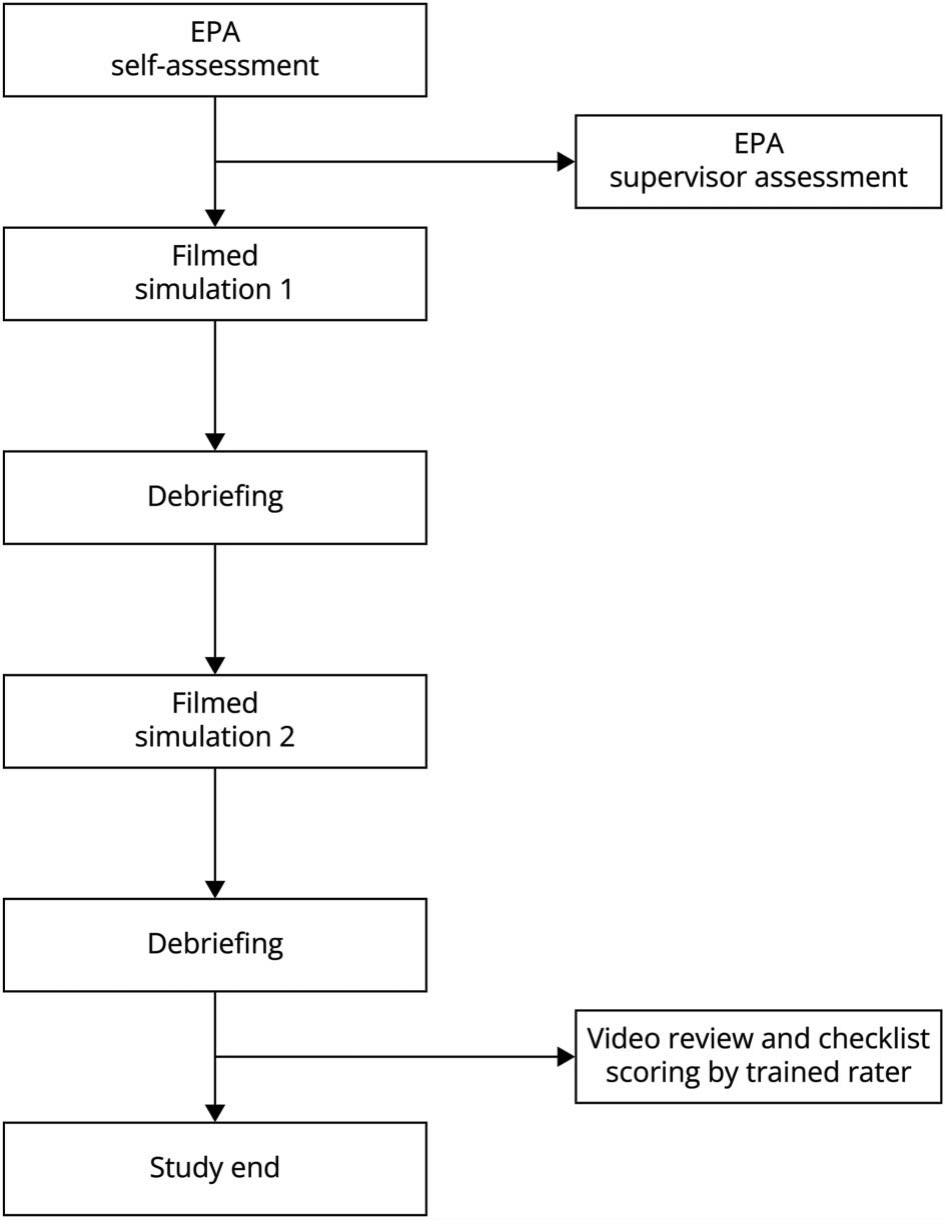
Study Flowchart EPA = entrustable professional activity.

The study protocol is illustrated in Figure 1. Participants completed an EPA self-assessment. Supervisors, including physicians and lead APPs where available, who were members of the study team also completed EPA-based assessments for each participant. The EPA-based assessors did not undergo specific training to complete the EPA-based assessments; however, all were familiar with EPA-based assessment through previous work developing EPAs for NCC APPs.^4^ Participants then completed 2 filmed simulations of medical and neurologic emergencies. Finally, a trained rater at each site watched the videos and scored each participant on the behavior checklist. The trained raters were the study team members who completed the EPA-based assessments.

### Simulation Scenario Development

Two simulated scenarios that each included 1 neurologic and 1 medical emergency were developed by the study team. The first scenario was a case of a patient with vasospasm in the context of aneurysmal subarachnoid hemorrhage (SAH) complicated by septic shock. The second scenario was a case of a patient with intracranial hypertension in the setting of acute traumatic brain injury leading to airway compromise. These emergencies were selected because management of vasospasm, traumatic brain injury, septic shock, and airway compromise were identified by a multi-institutional group of physicians and APPs as EPAs with a high level of agreement (>95% expert consensus) during a previous Delphi process.^4^ Furthermore, these situations could be replicated with high fidelity in the simulation environment at the participating institutions. The scenarios were developed iteratively by members of the study team, including NCC APPs, NCC fellows, and NCC staff physicians from all participating institutions. Two members of the study team were trained in simulation design at the Harvard Center for Medical Simulation (CMS).^14^ The scenarios were then reviewed by a subject matter expert (SME) at an outside institution, with feedback incorporated. The scenarios were pilot tested, with final adjustments made to the cases based on participant feedback and behavior before enrolling participants.

### Intervention

Each APP participated in the simulated scenarios alone. Before participating in the simulation, participants were “prebriefed” to introduce the reason for the activity, ensure participant confidentiality, and establish a fiction contract. A member of the study team playing the patient's nurse was in the room for each scenario. Each institution used a manikin and monitor, controlled by either a second member of the study team or a simulation operator. Institutions were not required to use the same manikin or monitor. Minimum manikin requirements included realistic mimicking of human anatomy, including a patent airway for intubation. Monitors were required to show heart rate, oxygen saturation, blood pressure, temperature, and 3-lead electrocardiogram (EKG). Each simulation was debriefed using the Debriefing with Good Judgment method by a debriefer trained at CMS.^15^

### Instrument Development

The EPA-based assessment questionnaire was developed iteratively by consensus of the investigators (eAppendix). To ensure survey items were derived from the construct of interest, they were based on core and nested NCC APP EPAs that had the highest (>95%) degree of expert consensus during a previous Delphi process (content validity).^4^ A generic supervision scale was used for assessment of each EPA, following best practices for entrustment supervision scale development (response process).^5^ This scale was selected because it was previously developed in this context as part of the aforementioned Delphi process to reflect the levels of entrustment a new NCC APP would be expected to progress through. The self-assessment questionnaire further included background questions on participant training history, practice setting, and work history.

The behavior checklist for each scenario was developed iteratively by consensus of the investigators based on relevant guideline recommendations and/or Emergency Neurological Life Support protocols, as available (eTables 1 and 2).^16-20^ Two SMEs at outside institutions then reviewed the checklists, and their feedback was incorporated. Finally, the checklists were pilot tested and revised before enrolling participants.

### Rater Training and Calibration

Each participant video was reviewed by 1 of 2 raters who were members of the study team. Before scoring any participant videos, each rater reviewed and scored 2 mock cases of each scenario. Participants in the mock cases were members of the study team portraying 1 participant with limited proficiency and 1 expert NCC APP. Interrater reliability was calculated based on these scores. Then, the study team then met to adjudicate discrepancies and to outline instructions to raters, specifying how each item should be scored during the study. Once the study team, including the raters, agreed on how to score each item, the raters were allowed to begin scoring participant videos.

### Outcomes

The primary outcome was the significance of the relationship between checklist-based performance in simulated medical and neurologic emergencies and supervisor-assessed overall level of entrustment among NCC APPs (relationship to other variables). For this comparison, only core and nested EPAs practiced in the simulation were included in the determination of overall entrustment. Secondary outcomes were the agreement between supervisor-assessed and self-assessed entrustment among NCC APPs. Post hoc exploratory analyses were conducted to assess the relationship between provider experience (in months) and supervisor-assessed entrustment, as well as correlation between checklist-based performance subscores and average supervisor scores on related nested EPAs.

### Statistical Analysis

Participant training history, practice setting, and work history are outlined with simple descriptive statistics. Interrater reliability for the rater calibration exercise was calculated using the kappa statistic, and Cronbach α was calculated for all responses to the EPA-based assessment questionnaire (internal structure). The association between EPA-based assessment and performance in simulated scenarios or experience was assessed using Spearman correlation. Differences between overall and individual EPA-based self-assessment and supervisor assessment were determined using the Kruskal-Wallis H test, followed by Dunn post hoc tests. Agreement between EPA-based self-assessment and supervisor assessment was assessed using weighted Kappa and visualized with an agreement chart. With the exception of the agreement chart (created using R version 4.4.2), statistical analysis was performed using SPSS version 29.0.2.0.

### Standard Protocol Approvals, Registrations, and Participant Consents

This study was approved by the Mass General Brigham, Boston University, and Emory University institutional review boards. All participants consented to participate in the research curriculum and to be filmed during the simulation.

### Data Availability

Anonymized data not published within this article will be made available by request from any qualified investigator.

## Results

Across the 5 institutions, 28 participants were enrolled. Details of participant training history, practice setting, and work history are provided in Table 2. In addition to EPA-based self-assessments and physician supervisor assessments, 17 participants also had an EPA-based assessment completed by an APP supervisor. Cronbach α for the EPA-based assessment was 0.987.

**Table 2:** Participant Characteristics

Participants	28
Training history	
Physician assistant (%)	6 (21.4)
Nurse practitioner (%)	22 (78.6)
Previous fellowship training (%)	2 (7.1)
Work experience	
Months of neurocritical care experience, median (IQR)	39 (12.5–86.5)
Previous advanced practice provider work experience (%)	5 (17.9)
Work setting	
Day shift (%)	18 (64.3)
Night shift (%)	2 (7.1)
Hybrid (%)	8 (28.6)

### Checklist and Rater Training

The final checklist included 43 behaviors across the 2 simulated scenarios. Raters agreed on 80 of 86 checklist items (93.0%) in the rater training exercise, corresponding to almost perfect agreement (κ = 0.822).

### EPA-Based Assessment and Performance in Simulated Scenarios

There was a significant positive correlation between performance in simulated scenarios and overall physician supervisor–assessed entrustment (r_s_ = 0.57, *p* = 0.002). There was no significant correlation between checklist-based performance and APP supervisor–assessed entrustment (r_s_ = −0.02, *p* = 0.952). There was a significant positive correlation between checklist-based performance on the intracranial hypertension, vasospasm, and sepsis-related items and average supervisor scores on the related nested EPAs (r_s_ = 0.59, *p* < 0.001; r_s_ = 0.38, *p* = 0.047; r_s_ = 0.43, *p* = 0.024). There was no significant correlation between checklist-based performance on the airway emergency–related items and average supervisor scores on the related nested EPA (r_s_ = 0.22, *p* = 0.333).

### EPA-Based Self-Assessment and Supervisor Assessment

There was no difference between overall EPA physician supervisor assessment, APP supervisor assessment, and self-assessment (5-point entrustment-supervision scale median [IQR], 4 [3–5] vs 4 [4–5] vs 4 [4-4], *p* = 0.598). Comparisons between physician supervisor, APP supervisor, and self-assessment of each core and nested EPA are shown in Table 3. Physician supervisor–assessed entrustment was higher than APP supervisor-assessed and self-assessed entrustment for stabilizing acute SAH (5 [4.5–5] vs 4 [4–4.5], *p* = 0.014; 5 [4.5–5] vs 4 [3–4], *p* < 0.001) and for identifying and managing status epilepticus (5 [4.5–5] vs 4 [4-4], *p* = 0.003; 5 [4.5–5] vs 4 [3–4], *p* < 0.001). Physician supervisor–assessed entrustment was lower than both APP supervisor–assessed and self-assessed entrustment for managing an airway (3 [3–4] vs 5 [4–5], *p* = 0.007; 3 [3–4] vs 4 [4–4.5], *p* = 0.020).

**Table 3:** Comparison Between EPA Supervisor Assessment and Self-Assessment

EPA	Physician	APP	Self	*p* Value (Kruskal-Wallis)	*p* Value (Dunn, self vs physician)	*p* Value (Dunn, self vs APP)	*p* Value (Dunn, physician vs APP)
Overall	4 (3–5)	4 (4–5)	4 (4–4)	0.598			
Core EPAs							
History, examination, and illness acuity	4 (4–5)	5 (4–5)	4 (4–5)	0.410			
Neuroimaging and neuromonitoring	4 (3–5)	4 (4–4)	4 (3–4)	0.629			
Neurologic emergency management	4 (3–5)	4 (4–5)	4 (3–4)	0.354			
Performing procedures	5 (2–5)	4 (4–4)	4 (3–4)	0.506			
Medical emergency management	4 (3–5)	4 (4–5)	4 (4–4)	0.203			
Communication	4 (4–5)	5 (4.5–5)	4 (4–5)	0.123			
Nested EPAs							
History and examination	5 (4–5)	5 (4.5–5)	4 (4–5)	0.185			
Malignant cerebral edema	4 (3–5)	4 (4–4)	4 (3–4)	0.605			
Elevated ICP and brain herniation	4 (3–5)	4 (4–4)	4 (3–4)	0.849			
ICH and IVH	4 (3–5)	4 (4–4.5)	4 (3–4)	0.528			
Acute SAH	5 (4.5–5)	4 (4–4.5)	4 (3–4)	0.002	<0.001	0.342	0.014
Vasospasm	4 (3.5–5)	4 (4–4)	4 (3–4)	0.321			
Status epilepticus	5 (4.5–5)	4 (4–4)	4 (3–4)	<0.001	<0.001	0.801	0.003
Shock	3.5 (3–4)	4 (4–4)	4 (3–4)	0.263			
Infection	3.5 (3–4.5)	4 (4–5)	4 (4–4)	0.150			
Respiratory failure	4 (4–4)	4 (3–4)	4 (3–4)	0.713			
Presenting patients	5 (4–5)	5 (5–5)	4 (4–5)	0.127			
Team communication	5 (4–5)	5 (5–5)	4 (4–5)	0.080			
Family/surrogate communication	4 (4–5)	5 (5–5)	4.5 (4–5)	0.057			
Escalating to fellow/attending	4 (4–5)	5 (5–5)	4 (4–5)	0.057			
Airway management	3 (3–4)	5 (4–5)	4 (4–4.5)	0.009	0.020	0.65	0.007

Abbreviations: APP = advanced practice provider; EPA = entrustable professional activity; ICH = intracerebral hemorrhage; ICP = intracranial pressure; IVH = intraventricular hemorrhage; SAH = subarachnoid hemorrhage.

**Figure 2:**
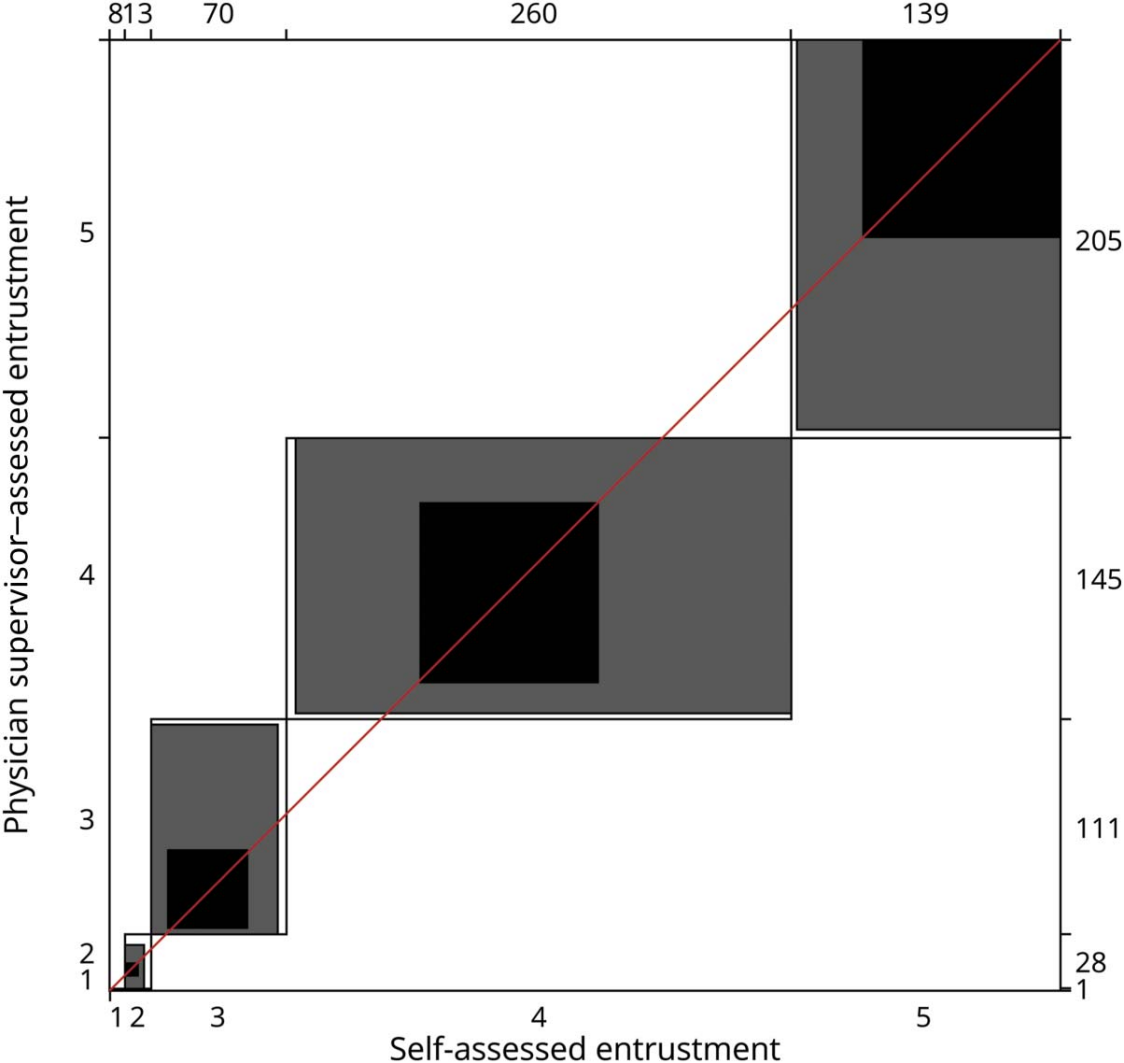
Agreement Chart of Matched Physician Supervisor–Assessed and Self-Assessed Entrustable Professional Activities Black squares represent perfect agreement, gray squares represent partial agreement, and white boxes represent maximum possible agreement. Numbers on the right vertical and top horizontal axes represent total number scores per level of entrustment.

Among 490 individual EPAs that were both self-assessed and assessed by a physician supervisor, there was substantial agreement (κ_w_ = 0.62, Figure 2). Among 322 individual EPAs that were self-assessed and assessed by an APP supervisor, there was fair agreement (κ_w_ = 0.38).

### EPA-Based Assessment and Experience

There was a significant positive correlation between participant experience and physician supervisor–assessed entrustment (r_s_ = 0.85, *p* < 0.001). There was no significant positive correlation between participant experience and APP supervisor–assessed entrustment (r_s_ = 0.04, *p* = 0.882).

## Discussion

In this study, physician supervisor EPA-based assessment was positively correlated with NCC APP performance in simulated scenarios, and there was substantial agreement between the physician supervisor's EPA-based assessment and APP's EPA-based self-assessment. Together, these findings suggest that EPA-based assessments may facilitate entrustment decisions for NCC APPs.

These findings have several practical implications. First, the correlation between physician supervisor assessment and simulation performance supports the validity of EPA-based assessment in this context, suggesting that it can help guide decisions about reducing supervision when a NCC APP has developed sufficient, specific competence. This competency-based medical education (CBME) model, in which clinicians progress to lower levels of supervision as they demonstrate competence, is particularly advantageous for APPs who may have significant heterogeneity in educational and training backgrounds before a first job.^21^ The educational needs of an acute care nurse practitioner with previous neurology nursing experience may be very different from those of a physician assistant who has completed a critical care fellowship. Use of EPA-based assessments could thus enable NCC APPs to complete orientation or attain supervisory responsibility for other APPs once they have demonstrated the requisite entrustment in the targeted EPAs, rather than in a time-based fashion.

While the correlation between APP entrustment and performance in NCC simulations was specifically evaluated in this study, the need for a standardized assessment method with supporting validity evidence reaches beyond this subspecialty and applies to all APPs who are new to practice. EPAs have previously been described for emergency medicine nurse practitioners, and development of EPAs for general neurology APPs is underway.^22^ EPAs could be developed for other groups of neurology APPs as a didactic framework and tool for assessment.

Furthermore, the appeal of implementing CBME is not unique to APP practice.^23^ EPA-based assessment could also inform time-variable transitions from training to practice for medical students and graduate medical trainees. EPA-based assessment is already in use in programs implementing CBME.^24^ Similar to EPAs, milestones, which are markers of progression toward a competency or subcompetency that are already in use in neurology training in the United States, may be a useful concept in CBME.^25,26^ However, unlike EPA-based assessment, milestone-based assessment has not been consistently associated with performance.^27^ Our finding that EPA-based assessment is correlated with performance in simulated scenarios highlights the potential benefit of this tool in CBME implementation.

We found substantial agreement between NCC APP self-assessed and physician supervisor–assessed entrustment. This agreement was stronger than that which has been reported previously in physician trainees.^10^ Aside from airway management, there were no EPAs for which participants self-assessed higher levels of entrustment compared with physicians. The alignment between areas of high entrustment and lower entrustment has implications for curricular development. It suggests that NCC APPs, who are adult learners, are well equipped to participate in the design of their own onboarding curricula, a key principle of andragogy theory.^28^ This approach could also inform their choices in self-directed CME, noting that airway management may be a relative blind spot.

That NCC APP self-assessed entrustment agreed with physician supervisor–assessed entrustment further has implications in APP education research. Specifically, this supports that EPA-based self-assessment could be used in survey-based research as a surrogate marker for performance when these data are unavailable. This would be a significant improvement over self-assessed confidence, which has been previously reported as a measure to describe impact of onboarding curricula for new neurology APPs but does not consistently correlate with performance.^25,29^ Data on available options for postgraduate APP training in neurology are largely limited to descriptions of single-center experiences.^26,30,31^ A readily measurable end point such as EPA-based self-assessment, which correlates with simulated performance, enables comparisons of the efficacy of existing postgraduate training models (such as the fellowship model vs on-the-job training), which will facilitate evidence-based decisions regarding which of these paradigms should be supported for APP training in neurology moving forward.

Unlike physician supervisor–assessed entrustment, APP supervisor–assessed entrustment did not correlate with performance in simulated scenarios. This may have been because there were fewer APP supervisors compared with physician supervisors who participated, yielding fewer comparisons and the possibility that the study was underpowered to detect a true effect. Alternatively, at each of the study sites, in contrast with physician supervisors, APP supervisors work alongside their colleagues but typically do not co-manage patients outside a brief preceptorship lasting several weeks or months during orientation. It was likely more difficult for APP supervisors to accurately assess their colleagues because they have less direct observation of their clinical performance.

There was a significant positive correlation between physician supervisor–assessed entrustment and participant experience, consistent with previous observations of the relationship between entrustment and experience.^7,32^ Others have reported this association as a form of construct validity, further supporting the use of EPA-based assessment. We highlight an alternate possibility—that there is potential for assessors to be biased by the experience of the person being assessed. Supervisors should be aware of this possibility when making entrustment decisions.

These findings have several limitations. All study sites were academic medical centers. It is unclear that these results would carry over to community-based practice. The sample size was relatively small, which may affect the robustness of the findings. The 2 simulated scenarios represent only a narrow portion of the NCC spectrum. As such, the results may not generalize to other domains of APP practice. Raters were not blinded to the identity of the participant, introducing risk of bias when scoring EPA-based assessments or behavior checklists (although there were some very junior APPs with high scores and more senior APPs with low scores). Internal consistency of the EPA-based assessment was very high, suggesting that some items may have been redundant and could be removed in future iterations. APPs in this study had on average 39 months of experience, and most worked on day shift where there is likely a higher degree of interaction with supervisors. This cohort of APPs thus may be better equipped to measure their proficiency and gaps in knowledge than APPs with very little experience or without such consistent exposure to a supervisor. Further research will be needed to evaluate the validity of EPA self-assessment in more junior APPs. Not all sites had a designated lead APP, and as above, it is possible that the study was underpowered to detect a significant correlation between APP supervisor and self-assessment. The entrustment supervision scale used for EPA-based assessment included only 5 steps, which may limit its sensitivity and reliability.^33^ While agreement in the rater training exercise was near perfect and additional steps for rater calibration were taken after calculation of interrater reliability, interrater reliability for the actual study data set was not established and may have been lower. Accurate assessment of entrustment relies on a sufficient number of assessments per EPA.^34^ Each participant in this study was evaluated by only 1 or 2 assessors. It is possible that, if additional EPA-based assessments were completed per participant, then the strength of the association between entrustment and checklist-based performance would have been higher. The primary “other” variable with which entrustment was compared (checklist-based performance) is not a perfect surrogate for real-life performance, which poses a threat to the validity of the comparison.

In conclusion, EPA-based physician supervisor assessment was positively correlated with NCC APP performance in simulated scenarios and should be considered as a tool for implementation of CBME. There was substantial agreement between EPA-based physician supervisor assessment and self-assessment, highlighting the potential for APPs to contribute to the planning of their onboarding and ongoing educational curricula.

## Glossary

APP: advanced practice provider
CBME: competency-based medical education
CMS: Center for Medical Simulation
EPA: entrustable professional activity
NCC: neurocritical care
SAH: subarachnoid hemorrhage
